# Altered perivascular spaces in subcortical white matter in Parkinson’s disease patients with levodopa-induced dyskinesia

**DOI:** 10.1038/s41531-024-00688-0

**Published:** 2024-03-28

**Authors:** Xingyue Cao, Caiting Gan, Heng Zhang, Yongsheng Yuan, Huimin Sun, Li Zhang, Lina Wang, Lian Zhang, Kezhong Zhang

**Affiliations:** Department of Neurology, The First Affiliated Hospital of Nanjing Medical University, Nanjing, 210029 China

**Keywords:** Parkinson's disease, Parkinson's disease

## Abstract

Dilated perivascular spaces (PVS) have emerged as a pathological hallmark in various neurological conditions, including Parkinson’s disease (PD). Levodopa-induced dyskinesia (LID), an intractable motor complication of PD, remains enigmatic regarding the distribution patterns of PVS. Our objective was to scrutinize the percent PVS (pPVS) changes within PD patients with LID (PD-LID). In total, 132 individuals were enrolled, including PD-LID (*n* = 42), PD patients without LID (PD-nLID, *n* = 45), and healthy controls (HCs, *n* = 45). Employing an automated approach for PVS quantification based on structural magnetic resonance imaging, we comprehensively evaluated total pPVS in subcortical white matter globally and regionally. A significant increase in global pPVS was observed in PD patients versus HCs, particularly evident in PD-LID relative to HCs. Within the PD-LID group, elevated pPVS was discerned in the right inferior frontal gyrus region (rIFG) (pars opercularis), contrasting with PD-nLID and HCs. Moreover, PD patients exhibited increased pPVS in bilateral superior temporal regions compared to HCs. Notably, pPVS in the rIFG positively correlated with dyskinetic symptoms and could well identify LID. Our findings unveiled PVS alternations in subcortical white matter in PD-LID at both global and regional levels, highlighting the increased pPVS in rIFG as a prospective imaging marker for LID.

## Introduction

Perivascular spaces (PVS), originally referred to as Virchow–Robin spaces, constitute fluid-filled compartments positioned between the basement membrane of penetrating blood vessels and the glia limitans formed by astrocytic endfeet processes^1,2^. These spaces share functional similarities with peripheral lymphatic vessels, as they maintain a contiguous connection with the subarachnoid space and assume a vital role in cerebral fluid dynamics and waste clearance^3,4^. While typically microscopic, PVS become discernible through magnetic resonance imaging (MRI) upon dilation, predominantly localizing within the basal ganglia (BG) and subcortical white matter^5^. Increased and dilated PVS are linked to aging, vascular risk factors, sleep disorders, and a variety of neurodegenerative diseases^4,6,7^.

Levodopa-induced dyskinesia (LID) stands as a refractory and incapacitating motor complication of Parkinson’s disease (PD). Principally manifested as involuntary chorea movements, it occurs in more than 50% of patients a decade after levodopa replacement treatment^8^. This greatly diminishes quality of life and escalates healthcare expenditures^9^. Currently, the intricate pathogenesis of LID remains incompletely elucidated. The classic LID model consists of two fundamental events at the striatal level, including insufficient endogenous dopamine production and storage due to severe nigrostriatal degeneration, and maladaptive synaptic plasticity triggered by intermittent stimulations of exogenous dopamine^10,11^. These events perturb neuronal firing and oscillatory patterns in the fronto-BG circuit, ultimately causing overactivation of the widespread motor network and exaggerated movements^12,13^.

The prominent feature of PD is the aberrant deposition of α-synuclein (α-Syn) throughout the brain^14^. PVS facilitate the elimination of neurotoxic proteins from the brain parenchyma^4,15,16^. Blocking meningeal lymphatic drainage, downstream of the PVS clearance system can lead to increased aggregation of α-Syn^17–19^. Neuroimaging studies have identified reduced diffusivity along PVS in PD patients, suggesting impaired PVS function in the context of PD^20,21^. Notably, in the BG, dilated and increased PVS corresponded with the severity of motor symptoms and cognitive deterioration^22,23^, while abnormal PVS alterations in the white matter were related to freezing of gait in PD^24^. Furthermore, researchers discovered that altered PVS was involved in α-Syn clearance obstruction, a key pathological mechanism in PD^25^. These cumulative findings compellingly suggested an interconnection between altered PVS and the pathogenesis of PD. However, PVS patterns in LID remain unexplored.

PD is now recognized as a multi-system disorder with considerable immune dysfunction and neuroinflammation^26^. The LID-related chronic levodopa therapy could interact with a previous PD-related inflammatory state, inducing glial cell activation and pro-inflammatory cytokine release^27^. It would further exacerbate inflammatory responses and PVS burden, since PVS are implicated in neuroinflammation^28–30^. Meanwhile, preclinical studies have widely reported an increase in pro-inflammatory cells and cytokines in LID rats^31–33^. Factors such as abnormal α-Syn accumulation, glial cell dysfunction, and blood–brain barrier breakdown all impede the integrity of the PVS, resulting in PVS obstruction and enlargement^18,19,34^. Therefore, we speculated that, based on PD lesions, PVS in LID might further expand abnormally. Exploring the anatomical distribution of PVS across different white matter regions may furnish novel insights into the mechanism of LID.

In this study, we utilized an advanced and automated segmentation approach based on MRI that specifically quantitates PVS volume in subcortical white matter. It provides a more objective evaluation, fewer human errors, and volumetric and subregional analyses of PVS compared to visual rating scales^34^. Our objective was to explore the global and regional PVS distribution in PD patients with LID (PD-LID), delineate the link between PVS and the severity of dyskinesia, and further comprehend its complicated pathophysiological mechanism underlying LID.

## Results

### Clinical characteristics and white matter hyperintensities (WMH) findings

Clinical characteristics and WMH findings of PD-LID, PD patients without LID (PD-nLID), and healthy controls (HCs) are shown in Table 1. No significant differences were found in age, sex, education level, vascular risk factors (hypertension, diabetes mellitus, hyperlipidemia, and smoking), and WMH findings (WMH volume and WMH number) among the three groups or between PD and HCs. Mini-Mental State Examination (MMSE) scores were significantly lower in PD versus HCs. In addition, PD-LID and PD-nLID matched well in terms of clinical characteristics, including age of onset, disease duration, Levodopa equivalent daily dose (LEDD), Unified Parkinson’s Disease Rating Scale part III (UPDRS III), Hoehn and Yahr stage (H-Y stage), and sleep disorders (Excessive daytime sleep and REM Sleep Behavior Disorder).

**Table 1:** Clinical characteristics and WMH findings of participants

	PD		HCs	*P* values	
	LID	nLID		PD-LID vs PD-nLID vs HCs	PD vs HCs
*n*	42	45	45	-	-
Clinical variables					
Age, y	60.6 ± 8.6	59.5 ± 8.1	62.6 ± 7.1	0.184^a^	0.086^b^
Sex, *N* male/female (% m/f)	22/20 (52.4/47.6)	25/20 (55.6/44.4)	26/19 (57.8/42.2)	0.879^c^	0.681^c^
Education, y	10.4 ± 3.0	10.2 ± 3.4	10.7 ± 3.6	0.793^d^	0.590^e^
Age at onset, y	53.1 ± 9.2	53.3 ± 8.3	-	0.878^e^	-
Disease duration, y	7.5 ± 5.0	6.2 ± 3.8	-	0.122^e^	-
LEDD, mg	765.7 ± 362.6	732.7 ± 364.5	-	0.583^e^	-
UPDRS III (off-phase)	33.1 ± 14.5	30.0 ± 14.9	-	0.248^e^	-
UPDRS III (on-phase)	20.3 ± 10.1	19.3 ± 11.8	-	0.358^e^	-
H-Y stage (off-phase)	2.4 ± 0.7	2.2 ± 0.7	-	0.148^e^	-
H-Y stage (on-phase)	2.0 ± 0.6	1.9 ± 0.6	-	0.193^e^	-
UDysRS	27.5 ± 17.7	-	-	-	-
MMSE	28.0 ± 1.7	28.2 ± 1.5	28.9 ± 1.1	0.043^d,*^	0.013^e,*^
Sleep Disorders					
RBD, *N* yes/no (%)	19/23 (45.2/54.8)	21/24 (46.7/53.3)	-	0.894^c^	-
EDS, *N* yes/no (%)	13/29 (31.0/69.0)	12/33 (26.7/73.3)	-	0.659^c^	-
Vascular risk factors					
Hypertension, *N* yes/no (%)	12/30 (28.6/71.4)	13/32 (28.9/71.1)	9/36 (20.0/80.0)	0.553^c^	0.277^c^
Diabetes mellitus, *N* yes/no (%)	4/38 (9.5/90.5)	5/40 (11.1/88.9)	3/42 (6.7/93.3)	0.809^f^	0.751^f^
Hyperlipidemia, *N* yes/no (%)	7/35 (16.7/83.3)	9/36 (20.0/80.0)	8/37 (17.8/82.2)	0.919^c^	0.931^c^
Smoking, *N* yes/no (%)	9/33 (21.4/78.6)	10/35 (22.2/77.8)	7/38 (15.6/84.4)	0.688^c^	0.390^c^
WMH findings					
WMH volume (mL)	3.9 ± 5.1	3.4 ± 4.8	3.4 ± 4.2	0.791^d^	0.797^e^
WMH number	13.3 ± 5.9	12.8 ± 7.6	12.9 ± 6.7	0.415^d^	0.660^e^

Variables are expressed as means ± standard error of the mean for the continuous variables and as *N* with % for the categorical variables.

*P* refers to the statistical significance between groups. Significance level: *P* < 0.05 (*).

*EDS* excessive daytime sleep, *HCs* healthy controls, *H-Y stage* Hoehn and Yahr stage, *LEDD* Levodopa equivalent daily dose, *LID* Levodopa-induced dyskinesia, *mL* milliliters, *MMSE* Mini-Mental State Examination, *PD* Parkinson’s disease, *RBD* REM Sleep Behavior Disorder, *UDysRS* Unified Dyskinesia Rating Scale, *UPDRS* Unified Parkinson’s Disease Rating Scale, *WMH* white matter hyperintensities.

^a^One-way analysis of variance.

^b^Two-sample *t*-test.

^c^Chi-squared test.

^d^Kruskal-Wallis test.

^e^Mann-Whitney test.

^f^Fisher’s exact test.

### PVS analysis

**Fig. 1:**
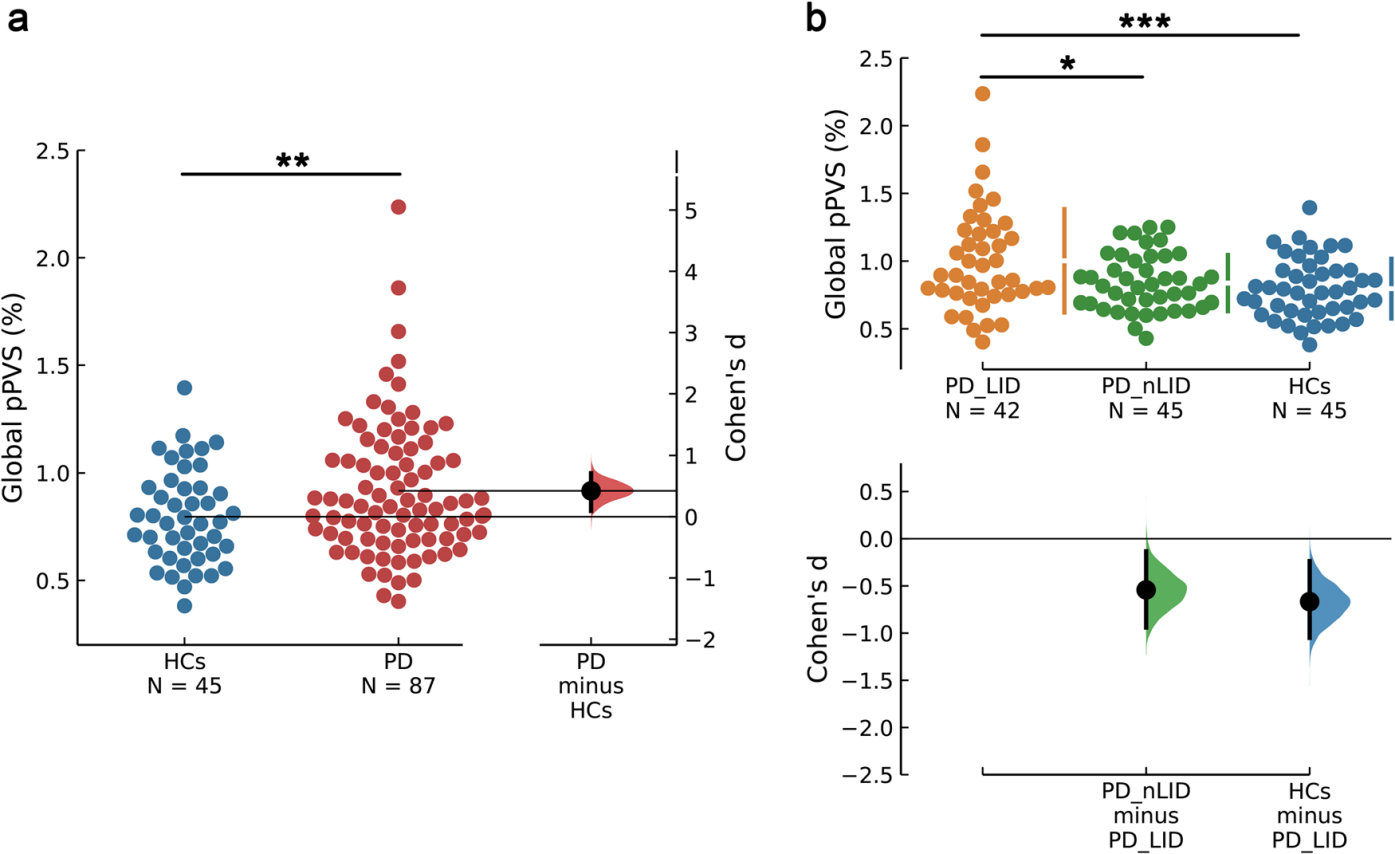
Global pPVS across subcortical white matter in PD-LID, PD-nLID and HCs. **a** The global pPVS in PD patients was significantly higher than in HCs (*P* = 0.009). **b** The global pPVS in PD-LID was significantly higher than in PD-nLID (*P* = 0.016) and HCs (*P* = 6.8 × 10^−4^). Swarm plots present global pPVS data points, and the second panels highlight the effect size (Cohen’s *d*) and present it as a bootstrap 95% confidence interval on an aligned axis. The larger the absolute value of Cohen’s *d*, the larger the effect size. *P* < 0.05 (*), *P* < 0.01 (**), and *P* < 0.001 (***) using ANCOVA followed by post-hoc analysis. Abbreviations: ANCOVA analysis of covariance, HCs healthy controls, LID Levodopa-induced dyskinesia, PD Parkinson’s disease, pPVS percent volume of perivascular spaces.

**Fig. 2:**
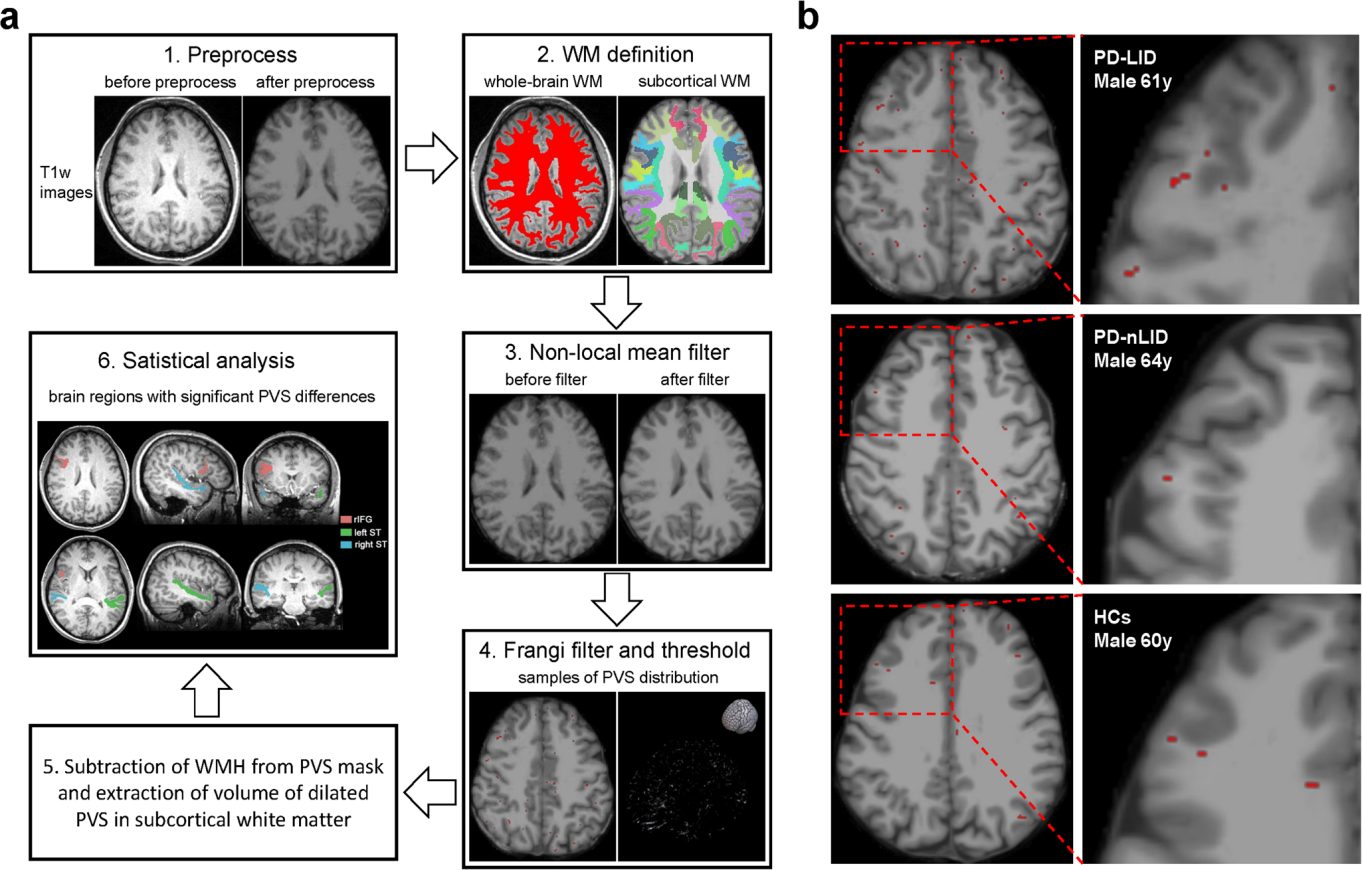
Schematic diagram showing the process of PVS quantification. **a** PVS quantification procedure : 1) preprocess, 2) white matter definition, 3) non-local mean filter, 4) frangi filter and threshold, 5) subtraction of WMH from PVS mask and extraction of volume of dilated PVS in subcortical white matter, 6) statistical analysis. **b** Sample differences of PVS distribution: T1w axial images of subcortical white matter PVS in PD-LID, PD-nLID, and HCs, with superimposed PVS illustrated in red. Abbreviations: HCs healthy controls, LID Levodopa-induced dyskinesia, PD Parkinson’s disease, PVS perivascular space, rIFG right inferior frontal gyrus region, ST superior temporal region, WM white matter, WMH white matter hyperintensities.

The global pPVS was significantly higher in PD relative to HCs (*F* = 7.099, *P* = 0.009 [Fig. 1a]). The analysis of covariance (ANCOVA) also showed a significant difference in global pPVS among PD-LID, PD-nLID, and HCs after adjusting for age, sex, education level, MMSE scores, WMH volume, WMH number, and intracranial volume (ICV) (*F* = 7.794, *P* = 6.5 × 10^−4^ [Fig. 1b]). According to post-hoc analysis, the global pPVS was significantly higher in PD-LID than in PD-nLID (*P* = 0.016) and HCs (*P* = 6.8 × 10^−4^). Sample differences in PVS distribution in subcortical white matter are shown in Fig. 2b.

**Fig. 3:**
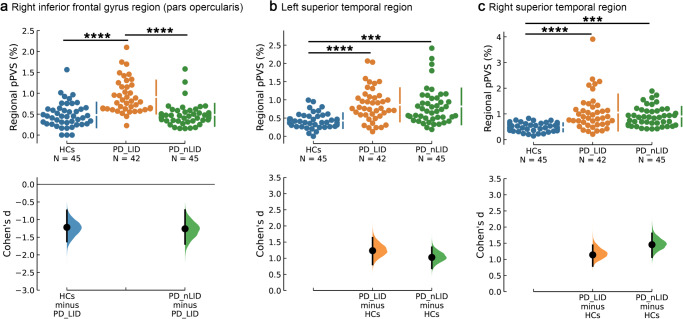
Regional pPVS in PD-LID, PD-nLID, and HCs. **a** The regional pPVS in PD-LID was significantly higher than in PD-nLID (*P* = 4.7 × 10^−8^) and HCs (*P* = 9.1 × 10^−8^) in the right inferior frontal gyrus region (pars opercularis). **b**, **c** The regional pPVS in PD-LID (Left: *P* = 1.8 × 10^−5^; Right: *P* = 7.8 × 10^−7^) and PD-nLID (Left: *P* = 1.2 × 10^−4^; Right: *P* = 4.4 × 10^−4^) in bilateral superior temporal region was significantly higher than in HCs. Swarm plots present regional pPVS data points, and the second panels highlight the effect size (Cohen’s *d*) and present it as a bootstrap 95% confidence interval on an aligned axis. The larger the absolute value of Cohen’s *d*, the larger the effect size. *P* < 0.001 (***) and *P* < 0.0001 (****) using ANCOVA followed by post-hoc analysis. Abbreviations: ANCOVA analysis of covariance, HCs healthy controls, LID Levodopa-induced dyskinesia, PD Parkinson’s disease, pPVS percent perivascular spaces.

**Fig. 4:**
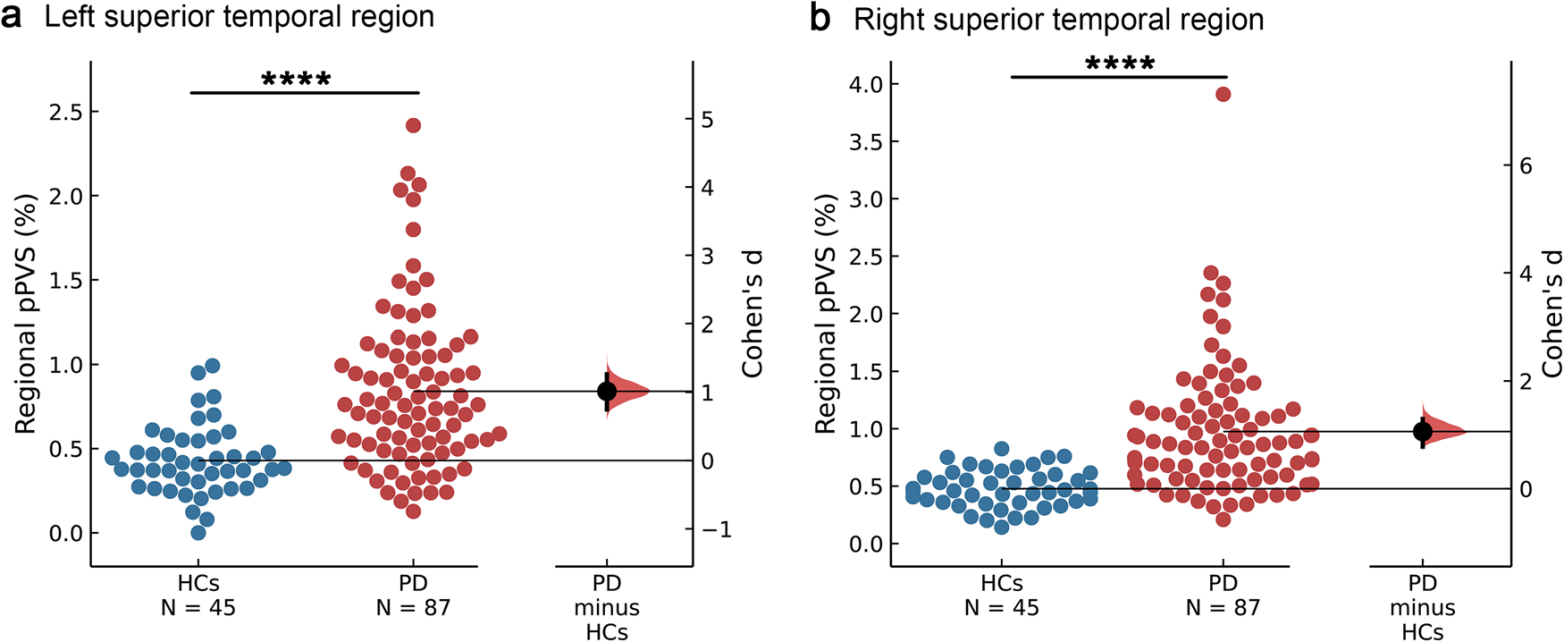
Regional pPVS in PD versus HCs. **a**, **b** The regional pPVS in PD in the bilateral superior temporal region was significantly higher than in HCs (Left: *P* = 1.0 × 10^−6^; Right: *P* = 5.2 × 10^−7^). Swarm plots present regional pPVS data points, and the second panels highlight the effect size (Cohen’s *d*) and present it as a bootstrap 95% confidence interval on an aligned axis. The larger the absolute value of Cohen’s *d*, the larger the effect size. *P* < 0.0001 (****) using ANCOVA. Abbreviations: ANCOVA analysis of covariance, HCs healthy controls, PD Parkinson’s disease, pPVS percent perivascular spaces.

The regional PVS comparisons were conducted in all 68 regions of the Desikan-Killiany atlas using ANCOVA followed by post-hoc analysis. Significant pPVS differences between PD-LID, PD-nLID, and HCs were observed in the right inferior frontal gyrus region (rIFG) (pars opercularis) (*F* = 23.872, *P* = 1.8 × 10^−9^ [Fig. 3a]) and bilateral superior temporal regions (ST) (Left: *F* = 13.369, *P* = 5.6 × 10^−6^ [Fig. 3b]; Right: *F* = 15.652, *P* = 8.9 × 10^−7^ Fig. 3c). Post-hoc analysis showed pPVS was significantly higher in PD-LID than in PD-nLID (*P* = 4.7 × 10^−8^) and HCs (*P* = 9.1 × 10^−8^) in the rIFG (Fig. 3a). The pPVS in PD-LID and PD-nLID in bilateral ST was significantly higher than in HCs (Left: *P*_PD-LID *vs* HCs_ = 1.8 × 10^−5^, *P*_PD-nLID vs HCs_ = 1.2 × 10^−4^ [Fig. 3b]; Right: *P*_PD-LID *vs* HCs_ = 7.8 × 10^−7^, *P*_PD-nLID *vs* HCs_ = 4.4 × 10^−4^ [Fig. 3c). The comparisons between PD and HCs also showed significant differences in bilateral ST (Left: *F* = 26.531, *P* = 1.0 × 10^−6^ [Fig. 4a]; Right: *F* = 28.095, *P* = 5.2 × 10^−7^ [Fig. 4b]). The schematic illustration of brain regions with significant regional pPVS differences is shown in Fig. 2a. All the above statistical analyses included age, sex, education level, MMSE scores, WMH volume, WMH number, and ICV as covariates, and the results were statistically significant after Bonferroni correction (*P* < 0.05/68).

### Correlation and receiver operating characteristic (ROC) curve analyses

**Fig. 5:**
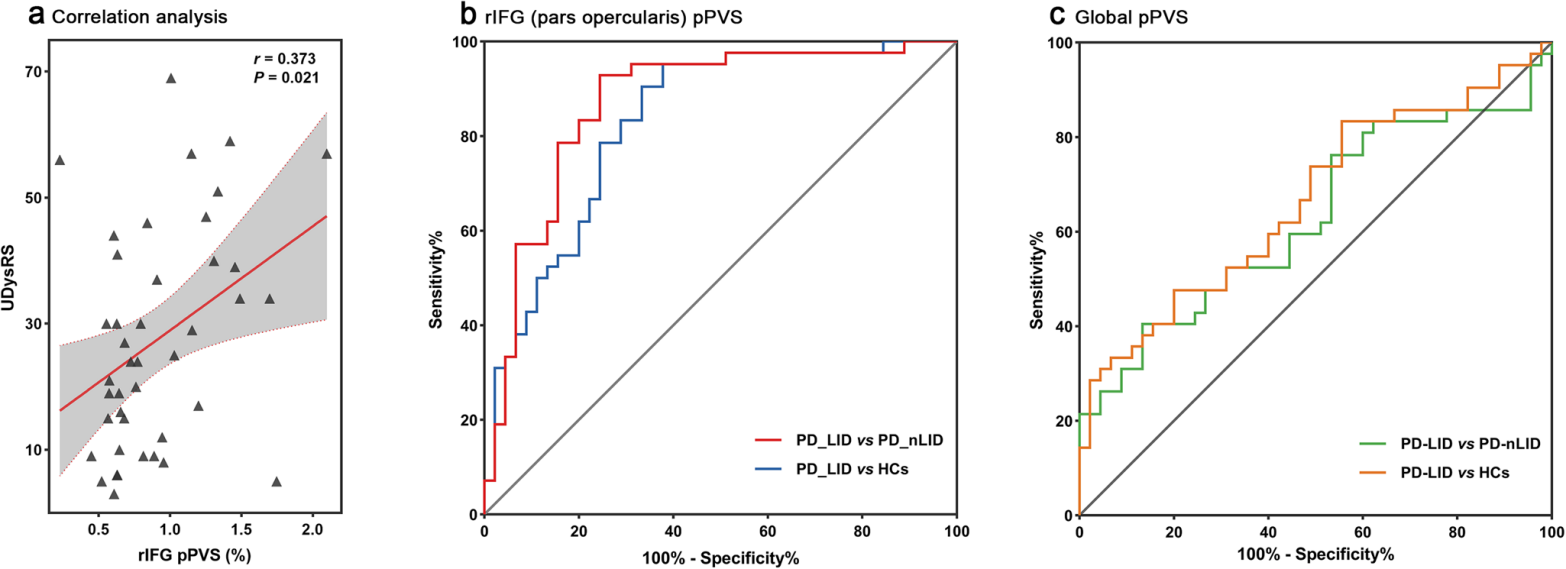
Correlation and ROC analyses. **a** The scatterplot indicated that there was a positive correlation between rIFG (pars opercularis) pPVS and UDysRS scores in PD patients with LID. **b**, **c** The graphs showed the results of the ROC analyses of rIFG (pars opercularis) pPVS and global pPVS. pPVS in rIFG had high efficiency for distinguishing PD-LID patients. HCs healthy controls, LID Levodopa-induced dyskinesia, PD Parkinson’s Disease, pPVS percent perivascular spaces, rIFG the right inferior frontal gyrus region, ROC receiver operating characteristic, UDysRS Unified Dyskinesia Rating Scale.

As illustrated in Fig. 5a, rIFG pPVS positively correlated with the Unified Dyskinesia Rating Scale (UDysRS) scores in PD-LID (*r* = 0.373, *P* = 0.021), while no other clinical or WMH characteristics correlated with rIFG pPVS or global pPVS in this subgroup. In the PD group, bilateral ST or global pPVS had no significant correlation with clinical characteristics. ROC analyses displayed that the area under the curve (AUC) of pPVS in the rIFG was 0.869 when separating PD-LID from PD patients without dyskinesia (95% confidence interval [CI]: 0.790–0.948, *P* < 0.001) and 0.830 when separating PD-LID patients from HCs (95% CI: 0.743–0.916, *P* < 0.001). Moreover, the AUC of the global pPVS was 0.628 when separating PD-LID patients from PD-nLID patients (95% CI: 0.508–0.747, *P* = 0.041) and 0.665 when separating PD-LID from HCs (95% CI: 0.550–0.779, *P* = 0.008). Details are shown in Table 2 and Fig. 5b, c.

**Table 2:** ROC analyses for distinguishing different groups

PVS findings	AUC	*P* value	95% CI	Sensitivity	Specificity	Cut-off point
*Global pPVS*						
Separating PD-LID from PD-nLID	0.628	0.041^*^	0.508–0.747	0.405	0.867	1.0587
Separating PD-LID from HCs	0.665	0.008^**^	0.550–0.779	0.833	0.444	0.7230
Separating PD-nLID from HCs	0.554	0.381	0.434–0.673	0.933	0.222	0.6065
*rIFG*_(pars opercularis)_ *pPVS*						
Separating PD-LID from PD-nLID	0.869	<0.001^***^	0.790–0.948	0.929	0.756	0.5508
Separating PD-LID from HCs	0.830	<0.001^***^	0.743–0.916	0.952	0.622	0.5198
Separating PD-nLID from HCs	0.505	0.939	0.383–0.627	0.800	0.356	0.3316

*P* refers to the statistical significance between groups. Significance level: *P* < 0.05 (*), *P* < 0.01 (**), *P* < 0.001 (***).

*AUC* area under the curve, *CI* confidence interval, *HCs* healthy controls, *LID* Levodopa-induced dyskinesia, *PD* Parkinson’s disease, *PVS* perivascular spaces, *pPVS* percent perivascular spaces, *rIFG* right inferior frontal gyrus region, *ROC* receiver operating characteristic.

## Discussion

Using a recently developed method of PVS-specific automated quantification based on MRI, the present study investigated the PVS distribution in PD-LID patients. A significant elevation in global and rIFG pPVS was observed in PD-LID compared to PD-nLID and HCs. PD patients, encompassing both PD-LID and PD-nLID, also exhibited heightened pPVS in bilateral ST relative to HCs. Furthermore, pPVS in rIFG was associated with dyskinetic symptoms and could act as an imaging marker for identifying PD-LID patients.

The long-held belief that the central nervous system lacked lymphatic drainage has been challenged by the recent discovery of the glymphatic system^3^. This system, comprised of the network of PVS pathways, allows cerebrospinal fluid (CSF) to enter the brain parenchyma along periarterial spaces via aquaporin-4 protein channels on astrocytic endfeet and exchange with interstitial fluid before being cleared by perivenous spaces^3^. CSF is eventually drained out of the brain through arachnoid granules or dural lymphatic vessels. As is widely acknowledged, PD is pathologically distinguished by widespread accumulation of α-Syn^14^. Proteins prone to intracellular accumulation, including α-Syn, are subject to glymphatic clearance^17,25^. Consistent with our findings, recent studies have reported increased PVS burden and reduced diffusivity along the PVS in PD patients^20–23^, suggesting impaired glymphatic function in PD. Research by Zou et al. revealed diminished glymphatic influx and heightened perivascular α-Syn aggregation in PD-like transgenic mice^17^. Upon ligating deep cervical lymph nodes, downstream of the PVS system, these mice presented increased deposition of α-Syn and worsened PD pathology^17^. Our study uncovered significant PVS dilation in PD patients and those concurrently experiencing LID. We hypothesized that the deteriorating α-Syn pathology might block PVS, which in turn could aggravate the impairment of PVS function. However, there are currently no reports in either animals or humans that the glymphatic system directly promotes α-Syn clearance. The development of in vivo tracking techniques for α-Syn is essential to fill this gap. Additionally, PVS also serves as an MRI indicator of brain inflammation^28–30^. Analogous to PD, LID is now acknowledged as a neuroinflammatory disorder involving substantial glial cell dysfunction and blood–brain barrier disruption, potentially disrupting PVS integrity and causing PVS enlargement^19,27,34^. Preclinical researches demonstrated astrogliosis and microglial activation, as well as increased pro-inflammatory cytokines in 6-hydroxydopamine-lesioned rats with LID^31–33^. This has been further confirmed by drug-related research that the administration of anti-inflammatory agents alleviated the motor symptoms of LID^35^. The increased global pPVS found in our study might be related to the deteriorated neuroinflammation in LID patients.

Regionally, we observed a notable rise in pPVS within rIFG in PD-LID patients. Extensive fiber connections exist between the rIFG and the superior frontal region, pre-supplementary motor areas, and temporal lobes^36^, all of which are integral components of the right-lateralized motor inhibition network. Motor inhibition denotes the capacity to suppress the inappropriate motor response. The rIFG plays a pivotal role in response inhibition during motor execution, akin to a “brake” for stopping or pausing responses^37^. It has been proven that inhibitory control was compromised in PD-LID patients which can be ameliorated by transcranial direct current stimulation of rIFG^38–40^. Previous MRI studies have identified LID-relevant structural and functional changes in IFG, such as volumetric increase and functional underactivation^41,42^. The detected pPVS increase in rIFG in LID patients might reveal the regional dysfunction of cerebral metabolism and impaired inhibitory control which would further contribute to involuntary movement^39^. Cerasa et al. proposed that the morphological remodeling of rIFG in LID was due to alternations of neuronal activity and plasticity induced by pulsatile and non-physiological administration of L-dopa^41^. The regional pPVS increase in LID also allows us to better comprehend previous anatomical changes found in the rIFG. To be specific, insufficient fluid drainage as well as excessive proliferation of glial cells associated with regional PVS dysfunction might be responsible for increased rIFG volume and thickness in LID^13^. We further discovered that the higher the pPVS in rIFG, the more severe the dyskinetic symptoms in LID. The level of pPVS might represent the extent of destruction in the rIFG which could generally underpin LID^37^ and could be proportional to the severity of dyskinesia. Additionally, pPVS in the rIFG had high efficiency for distinguishing PD-LID patients, and it could serve as an imaging marker for PD-LID patients.

Another regional finding was an increased pPVS in bilateral ST in PD patients, including those with and without LID. The ST is recognized for its involvement in memory and cognitive function, which is often impacted in PD patients^43^. Decreased functional activation in the ST was noted in PD relative to HCs^44^. Neurovascular decoupling in the ST has been linked to cognitive deterioration in individuals with PD^45^. The binding of neurogranin and α-Syn in the ST, a molecular process implicated in learning and memory, also decreased in PD along with the phosphorylation of neurogranin^46^. In our study, PD patients exhibited lower MMSE scores than HCs, and no significant associations were found between MMSE scores and pPVS in bilateral ST. It implied that the increase in pPVS in the ST might be an early sign of cognitive decline in PD patients.

Our investigation may offer a potential supplement for the diagnosis, prognosis and intervention of dyskinesia. The significant increase in PVS in LID and PD throughout subcortical white matter might indicate neurometabolic and hemodynamic abnormalities. Detailed analysis of PVS and glymphatic function might provide a more accurate biomarker for early diagnosis and differentiation of diseases. A more complete understanding of the relationship between PVS levels and the progression of LID may be achieved by continuous monitoring of PVS levels. It is worth exploring whether the sustained increase in PVS in the rIFG is closely related to the progression and worsening of LID. Mounting evidence has indicated that PVS dilation might be reversible, and enhancing glymphatic clearance could serve as a promising therapeutic target for neurodegenerative disorders. Methods such as good sleep hygiene and physical exercise have been documented to not just forestall but also decelerate the advancement of PD by enhancing glymphatic functionality, improving CSF flow, and naturally boosting cerebral waste elimination^47^. Moreover, there is also potential to improve the transport of drugs administered directly into the CSF space by regulating the glymphatic system^48^. Noteworthy is that PVS dilation is associated with vascular risk factors, of which hypertension is frequently reported^4,7^. Thus, good hypertension management may also aid in slowing down the PVS dilation and the progression of neurodegenerative diseases. Taken together, manipulating factors of the PVS system appear to be valid approaches for LID treatment.

It needs to be acknowledged that there are both concordance and inconsistencies between previous studies and our findings. Donahue et al. employed data processing methods nearly identical to ours, comparing global and regional pPVS differences among idiopathic PD, familial PD, HCs, and nonmanifest carriers^49^. Both studies identified higher levels of global and regional pPVS in PD patients compared to non-PD, particularly in specific regions such as bilateral ST, highlighting the relevance of elevated pPVS as a shared feature in PD pathology. Our study specifically addressed differences between PD subtypes, while Donahue et al. focused on familial PD and genetic carriers. They found that the medial orbitofrontal region shows key distinctions between all familial PD or pure leucine-rich repeat kinase 2 familial PD and nonmanifest carriers, as well as between PD and non-PD, emphasizing a potentially significant contribution of PVS to the pathophysiology of familial PD, particularly leucine-rich repeat kinase 2. Adding to this, evidence from early-stage PD showed that the PVS difference between PD and HCs primarily located in the BG^22^. We speculate that the differences may stem from varying disease duration of selected subjects and disparate PVS analysis approaches.

There are still some limitations to our study. First, although the quantitative method is more objective than rating scales, we only analyzed the PVS in subcortical white matter due to poor automated quantification in deep brain regions such as the BG. Methods such as manually delineating PVS boundary and applying 3D active contour segmentation are available options^22,50^. Second, due to the absence of a Freesurfer template for the centrum semiovale, a commonly explored and meaningful region, we refrained from conducting a pPVS analysis within this specific region. Third, being a cross-sectional study, it was unable to determine whether these PVS alterations were the cause or the consequence of LID. Our forthcoming efforts will center on further expanding our methodological knowledge to uncover quantitative changes in PVS within these region of interest and longitudinally investigating the causal relationship between PVS dysfunction and LID onset.

Collectively, we demonstrated PVS alterations in subcortical white matter in PD-LID patients, and it seemed to confirm the role of motor inhibition of rIFG in the occurrence of LID. PVS changes in rIFG might function as a potential imaging marker to assess dyskinetic symptoms and identify LID.

## Methods

### Participants and clinical assessments

Patients diagnosed with idiopathic PD according to the UK Parkinson’s Disease Society Brain Bank criteria^51^ were recruited from the Department of Neurology, the First Affiliated Hospital of Nanjing Medical University. Inclusion criteria were: (1) right handedness; (2) asymmetric onset; (3) no family history; (4) presence or absence of typical peak-dose LID after an acute levodopa administration assessed by two professional neurologists. Exclusion criteria were: (1) concurrent with any other neurodegenerative disorder, cerebrovascular disease, or cerebral trauma; (2) brain anatomical abnormalities; (3) cognitive impairment (MMSE score ≤ 24); (4) contraindications of MRI scans. Ultimately, 87 PD patients, including 42 patients with peak-dose LID (PD-LID) and 45 patients without dyskinesia (PD-nLID), and 45 age- and sex-matched HCs from surrounding communities were enrolled.

Clinical assessments were performed on each participant, including clinical histories and neurological evaluations. LEDD was calculated^52^. PD symptom severity was measured by the UPDRS III and H-Y stage during off-phase (withdrawal of dopaminergic drugs over 12 hours) and on-phase, respectively. The severity of dyskinetic symptoms in LID was assessed via the UDysRS^53^ during on-phase. The MMSE scale was applied to detect cognitive function.

Experimental protocols were approved by the ethics committee of the First Affiliated Hospital of Nanjing Medical University (Ethics No.2016-SRFA-094). All participants were informed and signed written consent before initiating the experiment.

### MRI acquisition

MRI scans were conducted during off-phase using a 3.0 T Siemens MAGNETOM Verio scanner (Siemens Medical Solutions, Germany) with an eight-channel head coil. Three-dimensional T1-weighted (T1w) anatomical images were obtained using the volumetric 3D magnetization-prepared rapid gradient-echo (MP-RAGE) sequence with the following parameters: repetition time [TR]/echo time [TE] = 1900/2.95 ms, flip angle [FA] = 9^°^, thickness = 1 mm, slices = 160, field of view [FOV] = 230 × 230 mm^2^, acquisition matrix = 256 × 256 and voxel size = 1 × 1 × 1 mm^3^. The fluid-attenuated inversion recovery (FLAIR) images were obtained with the following parameters: TR/TE = 8000/97 ms, FA = 150^°^, thickness = 5 mm, FOV = 230 × 230 mm^2^, acquisition matrix = 256 × 191 and echo train length = 16.

### WMH segmentation

WMH was automatically segmented by the lesion prediction algorithm (LPA)^54^ using the Lesion Segmentation Tool (LST v3.0.0) implemented in Statistical Parametric Mapping 12 (SPM12, https://www.fil.ion.ucl.ac.uk/spm/software/spm12/). FLAIR images were used for segmentation, and T1w images served as reference images during a co-registration step before segmentation. The LPA comprises a binary classifier founded on the logistic regression model. This model incorporates covariates, including a lesion belief map analogous to the one utilized in the lesion growth algorithm, along with a spatial covariate that accounts for voxel-specific changes in lesion probability. The preprocessing of raw FLAIR data was executed and lesion probability maps were estimated. Values of interest like total WMH volume and WMH number were extracted by thresholding individual lesion probability maps. WMH mask was constructed from original FLAIR images.

### PVS quantification

Firstly, T1w images were preprocessed and parcellated using the FreeSurfer (v7.2.0) software package^55^. Preprocessing process consisted of five steps, including motion correction, nonuniform intensity normalization, Talairach transformation, intensity normalization, as well as skull stripping. Parcellation was executed via the recon-all module employing an atlas-based method. White matter mask was derived from the Desikan-Killiany atlas^56^. ICV and white matter volume were calculated.

Subsequently, an advanced technique developed by ref. ^57^ was performed for PVS mapping and quantification, which mainly focused on subcortical white matter. Preprocessed T1w data were filtered using an adaptive non-local mean filtering technique^58^ implemented in the Advanced Normalization Tools software package. Notably, only high-frequency spatial sounds were filtered to maintain PVS voxels while reducing noise. This was accomplished by employing a filtering patch with a radius of 1 voxel, which reduces noise at the single-voxel level while preserving spatially repeating signal intensities. Then, we applied the Frangi filter^59^ module of the *Quantitative Imaging Toolkit*^60^ to T1w images. As recommended by ref. ^59^, default parameters were utilized, with *α* = 0.5, *β* = 0.5, and *c* set to half the maximum Hessian norm value. Frangi filter estimated vesselness measures at different scales and offered the highest likelihood. To optimize vessel inclusion, the scale was adjusted to a range of 0.1–5 voxels. This step produced a quantitative map of vesselness in the regions of interest, which represents the maximum across scales. The outputs across voxels comprise vesselness measured across a range of filter scales. To obtain the PVS mask, a vesselness threshold of 0.0002 was applied. To address potential mis-segmentation of PVS caused by WMH, the WMH mask was subtracted from the PVS mask. Firstly, a FLAIR-to-T1w image transformation matrix was generated by registering FLAIR images to preprocessed T1w images using the FMRIB Linear Image Registration Tool in FMRIB Software Library (FSL v6.0.4; https://www.fmrib.ox.ac.uk/fsl) with trilinear interpolation and six degrees of freedom. Then, the WMH mask was registered to the T1w image space by applying the FLAIR-to-T1w image transformation matrix. Subsequently, the WMH mask was subtracted from the PVS mask to ensure that the percent PVS calculations were based on normal white matter volumes. Finally, PVS volume and spatial distribution were calculated. Small components (<5 voxels) were excluded from automated counting to minimize noise contribution^57^.

### Statistical analysis

Quantitative data for clinical characteristics and WMH imaging findings were analyzed with either one-way analysis of variance or the Kruskal-Wallis test among three groups and the two-sample *t*-test or the Mann-Whitney test between two groups based on Shapiro-Wilk normality test results. The Chi-squared test or Fisher’s exact test was performed for the comparison of categorical data.

PVS comparisons were performed at the global and regional levels, respectively. For the global level, the volume of total subcortical white matter PVS was normalized to total subcortical white matter volume as the global percent volume of PVS (pPVS). Subsequently, regional pPVS was calculated as the volume of regional white matter PVS divided by the regional white matter volume in 68 subregions of the Desikan-Killiany atlas. The global and regional pPVS differences between PD-LID, PD-nLID, and HCs were initially compared, followed by the comparisons between PD and HCs. ANCOVA was applied for PVS analysis, adjusting for age, sex, education level, MMSE score, WMH volume, WMH number, and ICV. Multiple comparison correction was performed using Bonferroni correction. The unpaired Cohen’s *d* effect size was calculated to estimate the mean differences in pPVS. In order to calculate the 95% CI of the Cohen’s *d* effect size, 5000 bootstrap resamples were taken, bias corrected, and accelerated^61^.

Partial correlation analyses were employed to evaluate the associations between significant pPVS alterations in PD-LID versus PD-nLID and clinical variables, as well as WMH imaging findings in the PD-LID group. Age, sex, education level, and ICV were included as covariates. The associations between significant pPVS alterations and clinical characteristics were also analyzed in the PD group. The significant pPVS alterations between PD-LID and PD-nLID were further measured by ROC analyses to estimate whether they could be identified as LID recognition features. Optimal cut-off value was chosen by maximizing Youden’s index, and the specificity, sensitivity, and AUC were provided.

A *P* value < 0.05 (two-tailed) was considered statistically significant. All statistical analyses were performed using SPSS (version 26; IBM Corp., Armonk, NY, USA).

### Reporting summary

Further information on research design is available in the Nature Research Reporting Summary linked to this article.

### Supplementary information

Reporting Summary

## Data Availability

The data supporting the findings of this study are not publicly available due to privacy concerns for research participants. However, they can be shared upon reasonable request, such as reproducibility of research or external validation, by contacting the corresponding author with institutional review board approval. Restrictions may be applied to sensitive data for privacy preservation.
