# Supplementary material for: Altered perivascular spaces in subcortical white matter in Parkinson’s disease patients with levodopa-induced dyskinesia
Source: NPJ Parkinsons Dis. 2024 Mar 28;10:71. doi: 10.1038/s41531-024-00688-0 (PMC10978930; doi:10.1038/s41531-024-00688-0)
Supplement: Supplementary file 1 — Reporting Summary [file 41531_2024_688_MOESM1_ESM.pdf]

nature portfolio

Corresponding author(s): NPJPARKD-02255

Last updated by author(s): 2023/09/04

Reporting Summary

Nature Portfolio wishes to improve the reproducibility of the work that we publish. This form provides structure for consistency and transparency in reporting. For further information on Nature Portfolio policies, see our Editorial Policies and the Editorial Policy Checklist.

Statistics

For all statistical analyses, confirm that the following items are present in the figure legend, table legend, main text, or Methods section.

n/a	Confirmed	
☐	☒	The exact sample size (*n*) for each experimental group/condition, given as a discrete number and unit of measurement
☐	☒	A statement on whether measurements were taken from distinct samples or whether the same sample was measured repeatedly
☐	☒	The statistical test(s) used AND whether they are one- or two-sided<br>*Only common tests should be described solely by name; describe more complex techniques in the Methods section.*
☐	☒	A description of all covariates tested
☐	☒	A description of any assumptions or corrections, such as tests of normality and adjustment for multiple comparisons
☐	☒	A full description of the statistical parameters including central tendency (e.g. means) or other basic estimates (e.g. regression coefficient) AND variation (e.g. standard deviation) or associated estimates of uncertainty (e.g. confidence intervals)
☐	☒	For null hypothesis testing, the test statistic (e.g. *F*, *t*, *r*) with confidence intervals, effect sizes, degrees of freedom and *P* value noted<br>*Give P values as exact values whenever suitable.*
☒	☐	For Bayesian analysis, information on the choice of priors and Markov chain Monte Carlo settings
☐	☒	For hierarchical and complex designs, identification of the appropriate level for tests and full reporting of outcomes
☐	☒	Estimates of effect sizes (e.g. Cohen's *d*, Pearson's *r*), indicating how they were calculated

Our web collection on statistics for biologists contains articles on many of the points above.

Software and code

Policy information about availability of computer code

Data collection	No custom algotithms or software were used.
Data analysis	1. The PVS quantification was performed using the Freesurfer, ANTs and QIT softwares.<br>2. The WMH segmentation was performed using the LST implemented in SPM8.<br>3. Statistical analyses were conducted with SPSS Statistics software.<br>4. Statistical plots were generated by Python, GraphPad Prism and Photoshop.

For manuscripts utilizing custom algorithms or software that are central to the research but not yet described in published literature, software must be made available to editors and reviewers. We strongly encourage code deposition in a community repository (e.g. GitHub). See the Nature Portfolio guidelines for submitting code & software for further information.

Data

Policy information about availability of data

All manuscripts must include a data availability statement. This statement should provide the following information, where applicable:

- Accession codes, unique identifiers, or web links for publicly available datasets
- A description of any restrictions on data availability
- For clinical datasets or third party data, please ensure that the statement adheres to our policy

The data that support the findings of this study are available from the corresponding author upon reasonable request.

Research involving human participants, their data, or biological material

Policy information about studies with human participants or human data. See also policy information about sex, gender (identity/presentation), and sexual orientation and race, ethnicity and racism.

Reporting on sex and gender	We enrolled 42 PD patients with LID, 45 PD patients without LID and 45 health controls. The incidence of PD is about 1.7%, and LID occurred in 50% PD patients after 10 years of levodopa treatment. We think our sample size are sufficient.
Reporting on race, ethnicity, or other socially relevant groupings	The participants were grouped according to their own disease status.
Population characteristics	For PD-LID patients, 20 were females and 22 were males. Their average age was 60.6 and all of them were treated with dopamine drugs. For PD-nLID patients, 20 were females and 25 were males. Their average age was 59.5 and all of them were treated with dopamine drugs. For health controls, 19 were females and 26 were males. Their average age was 62.6 and none of them were treated with dopamine drugs.
Recruitment	Patients diagnosed with idiopathic PD according to the UK Parkinson's Disease Society Brain Bank criteria were recruited from the Department of Neurology, the First Affiliated Hospital of Nanjing Medical University. Inclusion criteria were: (1) right handedness; (2) asymmetric onset; (3) no family history; (4) presence or absence of typical peak-dose LID after an acute levodopa administration assessed by two professional neurologists. Exclusion criteria were: (1) concurrent with any other neurodegenerative disorder, cerebrovascular disease, or cerebral trauma; (2) brain anatomical abnormalities; (3) cognitive impairment (MMSE score ≤ 24); (4) contraindications of MRI scans. Ultimately, 87 PD patients, including 42 patients with peak-dose LID (PD-LID) and 45 patients without dyskinesia (PD-nLID), and 45 age- and sex-matched HCs from surrounding communities were enrolled.
Ethics oversight	This study was approved by the ethics committee of the First Affiliated Hospital of Nanjing Medical University.

Note that full information on the approval of the study protocol must also be provided in the manuscript.

Field-specific reporting

Please select the one below that is the best fit for your research. If you are not sure, read the appropriate sections before making your selection.

☒ Life sciences ☐ Behavioural & social sciences ☐ Ecological, evolutionary & environmental sciences

For a reference copy of the document with all sections, see nature.com/documents/nr-reporting-summary-flat.pdf

Life sciences study design

All studies must disclose on these points even when the disclosure is negative.

Sample size	We enrolled 42 PD-LID patients, 45 PD-nLID patients and 45 health controls. The incidence of Parkinson's disease is about 1.7%, and LIDs occurred in 50% PD patients after 10 years of levodopa treatment. We think our sample size are sufficient.
Data exclusions	Patients with any other neurological or psychiatric diseases except for PD were excluded. Healthy controls had no history of neurological or psychiatric disease and no family history of PD or related neurodegenerative disorders.
Replication	All attempts at replication were successful.
Randomization	PD-LID, PD-nLID and healthy control groups were age- and sex- matched. There were no significant differences between the three groups in age, sex, education, prevalence of vascular risk factors (hypertension, hyperlipidemia, diabetes mellitus and smoking), sleep disorders, or WMH findings.
Blinding	Our study aimed to study the PVS alternations of PD-LID patients. And the participants were grouped according to their own disease status.

Behavioural & social sciences study design

All studies must disclose on these points even when the disclosure is negative.

Study description	
Research sample	
Sampling strategy	
Data collection	

Timing

Data exclusions

Non-participation

Randomization

Ecological, evolutionary & environmental sciences study design

All studies must disclose on these points even when the disclosure is negative.

Study description

Research sample

Sampling strategy

Data collection

Timing and spatial scale

Data exclusions

Reproducibility

Randomization

Blinding

Did the study involve field work? ☐ Yes ☐ No

Field work, collection and transport

Field conditions

Location

Access & import/export

Disturbance

Reporting for specific materials, systems and methods

We require information from authors about some types of materials, experimental systems and methods used in many studies. Here, indicate whether each material, system or method listed is relevant to your study. If you are not sure if a list item applies to your research, read the appropriate section before selecting a response.

Materials & experimental systems

n/a	Involved in the study
☒	☐ Antibodies
☒	☐ Eukaryotic cell lines
☒	☐ Palaeontology and archaeology
☒	☐ Animals and other organisms
☒	☐ Clinical data
☒	☐ Dual use research of concern
☒	☐ Plants

Methods

n/a	Involved in the study
☒	☐ ChIP-seq
☒	☐ Flow cytometry
☐	☒ MRI-based neuroimaging

Antibodies

Antibodies used

Validation

Eukaryotic cell lines

Policy information about cell lines and Sex and Gender in Research

Cell line source(s)

Authentication

Mycoplasma contamination

Commonly misidentified lines
(See ICLAC register)

Palaeontology and Archaeology

Specimen provenance

Specimen deposition

Dating methods

☐ Tick this box to confirm that the raw and calibrated dates are available in the paper or in Supplementary Information.

Ethics oversight

Note that full information on the approval of the study protocol must also be provided in the manuscript.

Animals and other research organisms

Policy information about studies involving animals; ARRIVE guidelines recommended for reporting animal research, and Sex and Gender in Research

Laboratory animals

Wild animals

Reporting on sex

Field-collected samples

Ethics oversight

Note that full information on the approval of the study protocol must also be provided in the manuscript.

Clinical data

Policy information about clinical studies
All manuscripts should comply with the ICMJE guidelines for publication of clinical research and a completed CONSORT checklist must be included with all submissions.

Clinical trial registration

Study protocol

Data collection

Outcomes

Dual use research of concern

Policy information about dual use research of concern

Hazards

Could the accidental, deliberate or reckless misuse of agents or technologies generated in the work, or the application of information presented in the manuscript, pose a threat to:

No	Yes	
☐	☐	Public health
☐	☐	National security
☐	☐	Crops and/or livestock
☐	☐	Ecosystems
☐	☐	Any other significant area

Experiments of concern

Does the work involve any of these experiments of concern:

No	Yes	
☐	☐	Demonstrate how to render a vaccine ineffective
☐	☐	Confer resistance to therapeutically useful antibiotics or antiviral agents
☐	☐	Enhance the virulence of a pathogen or render a nonpathogen virulent
☐	☐	Increase transmissibility of a pathogen
☐	☐	Alter the host range of a pathogen
☐	☐	Enable evasion of diagnostic/detection modalities
☐	☐	Enable the weaponization of a biological agent or toxin
☐	☐	Any other potentially harmful combination of experiments and agents

Plants

Seed stocks

Novel plant genotypes

Authentication

ChIP-seq

Data deposition

☐ Confirm that both raw and final processed data have been deposited in a public database such as GEO.

☐ Confirm that you have deposited or provided access to graph files (e.g. BED files) for the called peaks.

Data access links
May remain private before publication.

Files in database submission

Genome browser session
(e.g. UCSC)

Methodology

Replicates

Sequencing depth

Antibodies

Peak calling parameters

Data quality

Software

Flow Cytometry

Plots

Confirm that:

☐ The axis labels state the marker and fluorochrome used (e.g. CD4-FITC).

☐ The axis scales are clearly visible. Include numbers along axes only for bottom left plot of group (a 'group' is an analysis of identical markers).

☐ All plots are contour plots with outliers or pseudocolor plots.

☐ A numerical value for number of cells or percentage (with statistics) is provided.

Methodology

Sample preparation

Instrument

Software

Cell population abundance

Gating strategy

☐ Tick this box to confirm that a figure exemplifying the gating strategy is provided in the Supplementary Information.

Magnetic resonance imaging

Experimental design

Design type: We focused on structural MRI data.

Design specifications: We focused on structural MRI data.

Behavioral performance measures: Patients were evaluated by clinical assessments, including UPDRS-III, H&Y stage, UDysRS, MMSE, sleep disorders, and vascular risk factors.

Acquisition

Imaging type(s): structural

Field strength: 3.0 T

Sequence & imaging parameters: Three-dimensional T1-weighted (T1w) anatomical images were obtained using the volumetric 3D magnetization-prepared rapid gradient-echo (MP-RAGE) sequence with the following parameters: repetition time [TR]/echo time [TE] = 1900/2.95 ms, flip angle [FA] = 9°, thickness = 1 mm, slices = 160, field of view [FOV] = 230 × 230 mm2, acquisition matrix = 256 × 256 and voxel size = 1 × 1 × 1 mm3. The fluid-attenuated inversion recovery (FLAIR) images were obtained with the following parameters: TR/TE = 8000/97 ms, FA = 150°, thickness = 5 mm, FOV = 230 × 230 mm2, acquisition matrix = 256 × 191 and echo train length = 16.

Area of acquisition: whole brain scan

Diffusion MRI ☐ Used ☒ Not used

Preprocessing

Preprocessing software: Freesurfer, ANTs, QIT, Matlab, SPM8, LST

Normalization: For the global level, the volume of total subcortical white matter PVS was normalized to total subcortical white matter

Normalization	volume as the global percent volume of PVS (pPVS). Regional pPVS was calculated as the volume of regional white matter PVS divided by the regional white matter volume in 68 subregions of the Desikan-Killiany atlas.
Normalization template	total subcortical white matter volume and regional white matter volume
Noise and artifact removal	Small components (<5 voxels) were excluded from automated counting to minimize noise contribution.
Volume censoring	QIT

Statistical modeling & inference

Model type and settings	Case controlled study
Effect(s) tested	have used ANCOVA analysis

Specify type of analysis: ☒ Whole brain ☐ ROI-based ☐ Both

Statistic type for inference (See Eklund et al. 2016)	cluster-wise
Correction	Bonferroni correction

Models & analysis

n/a	Involved in the study
☒	☐ Functional and/or effective connectivity
☒	☐ Graph analysis
☒	☐ Multivariate modeling or predictive analysis

Functional and/or effective connectivity	
Graph analysis	
Multivariate modeling and predictive analysis	
